# Rickettsia conorii infection with fatal complication

**DOI:** 10.4322/acr.2021.392

**Published:** 2022-07-18

**Authors:** Sanjoli Chugh, Priyanka Kumari, Shriya Goel, Manisha Biswal, Inderpaul Singh Sehgal, Aravind Sekar

**Affiliations:** 1 Post Graduate Institute of Medical Education and Research, Department of Histopathology, Chandigarh, India; 2 Post Graduate Institute of Medical Education and Research, Department of Pulmonary Medicine, Chandigarh, India; 3 Post Graduate Institute of Medical Education and Research, Department of Medical Microbiology, Chandigarh, India

**Keywords:** Autopsy, Fatal Outcome, Fever, Rickettsia Infections, Vasculitis

## Abstract

Rickettsial diseases (RD) are a group of endotheliotropic infectious diseases caused by different species of genera *Rickettsia*. RD are not an uncommon disease and may be misdiagnosed during the evaluation of acute febrile illness due to a lack of reliable serological marker and diagnostic culture methods. Clinical manifestation of RD varies from febrile illness with rashes and myalgia to fatal complications such as shock and respiratory failure. We describe a case of a young male who presented initially with acute febrile illness, followed by shock and respiratory failure, and unfortunately succumbed to death. A post-mortem examination showed histological features of endotheliotropic infection, such as interstitial / perivascular edema in various organs and noncardiogenic pulmonary edema (suggesting increased vascular permeability) and evidence of vasculitis in the lung, liver, and intestines. Molecular studies performed from lung, liver, and kidney tissue confirm the diagnosis of spotted fever group rickettsial disease due to *Rickettsia conorii*.

## INTRODUCTION

Rickettsial diseases are caused by a varied group of obligate intracellular bacteria belonging to the Genus *Rickettsia* with a propensity to infect endothelial cells mainly.^1^ They are transmitted by infected hematophagous arthropods such as ticks, mites, flea, and lice. Spotted fever and typhus groups are two broad subgroups of rickettsial diseases. *R. conorii* causing spotted fever group and *R. typhi* causing murine typhus are now known to occur in India.^2^

The major clinical presentation of rickettsial diseases is an acute febrile illness with or without rash.^3^ The documented prevalence of RD in adults' febrile illness cases was 12.5% in southeast Asian countries.^4^ The disease's clinical severity depends on host-related factors such as genetic polymorphism and the species, quantity, and virulence of infected organisms. It may vary from fever associated with chills, myalgia, and jaundice to life-threatening events such as hypovolemic shock, severe respiratory failure, acute kidney injury, seizures, and coma. In the present era, a case fatality rate of Rocky Mountain spotted fever caused by *R. rickettsii* varies from 3 to 40%.^5^ Higher mortality was reported in Latin America due to more virulent strains. The documented case fatality rate of *R. prowazekii* infection is 15%. We describe clinical features and autopsy findings in a patient of spotted fever group RD, diagnosed during post-mortem evaluation in a young male who presented with acute febrile illness of unknown etiology.

## CASE REPORT

A 26-year-old man was referred to the emergency outpatient department for low blood pressure. He has been presenting high-grade fever (39,4 °C) over the last four days associated with chills, myalgias, calf pain, joint pain, and dull aching abdominal pain. He had a history of shortness of breath for one day and rashes on the lower limbs. There was no history of cough, nausea, vomiting, diarrhea, chest pain, palpitations, orthopnea, or any bleeding manifestations. There were no associated comorbidities.

On examination, he was conscious and well-oriented to time, place, and person. His pulse rate was 130/min and regular, respiratory rate 45/min, blood pressure 90/60 mmHg on noradrenaline, and SpO_2_ was 82% on 50% FiO_2_. A non-blanchable petechial rash was observed over bilateral hands and feet. An eschar was seen in the right groin region. The liver was enlarged and palpable up to 3 cm below the right costal margin with a smooth outline on abdominal examination. There was no splenomegaly or free fluid. An abdomen ultrasound showed normal-echotexture hepatomegaly with a span of 17.5 cm (RR;<16 cm). Both kidneys showed raised cortical echogenicity. Sinus tachycardia was observed in the electrocardiogram. Serology workup for dengue, leptospirosis, hepatitis B, and C was negative. The smear for malarial parasite was negative.

The laboratory findings on admission showed a hemoglobin 13.7 gm/dL (reference range [RR]; 13.8-17.2 gm/dL); total leukocyte count of 19,500 cell/mm^3^ (RR; 4,500-11,000 cells/mm^3^); platelet count of 21x10^3^ cells/mm^3^ (RR;150-450x103 cells/mm^3^); serum creatinine 1.8 mg/dL (RR;0.8-1.2 mg/dL); urea 100 mg/dl (RR; 6-24 mg/dl); AST/ALT-172/189U/L (RR; 7-55/8-48 U/L); PT/APTT-18/ 50 seconds (RR;10-12/30-40 seconds); INR-1.33 (RR;<1.1); CKMB-201 ng/ml (RR-26-192 ng/ml). The urinalysis was normal.

The possibility of tropical illness was considered and treated with intravenous ceftriaxone, doxycycline, and vasopressors. Because of persistent shock and respiratory distress, he was shifted to the respiratory intensive care unit and intubated. A second inotrope was added. Despite all supportive measures, his vitals declined, and he went into asystole. The resuscitation was unsuccessful and could not be revived. The patient was hospitalized for one and a half day.

## AUTOPSY FINDINGS

With due consent from the deceased relatives, a partial autopsy was performed without examining the brain 5 hours after death. Evisceration was done in toto with a midline thoracoabdominal incision. On the opening of the serous cavities, the peritoneal cavity yielded 700 ml of clear to reddish fluid, the pericardial cavity yielded 100ml of clear fluid, and the pleural cavity was within the normal limit.

**Figure 1:**
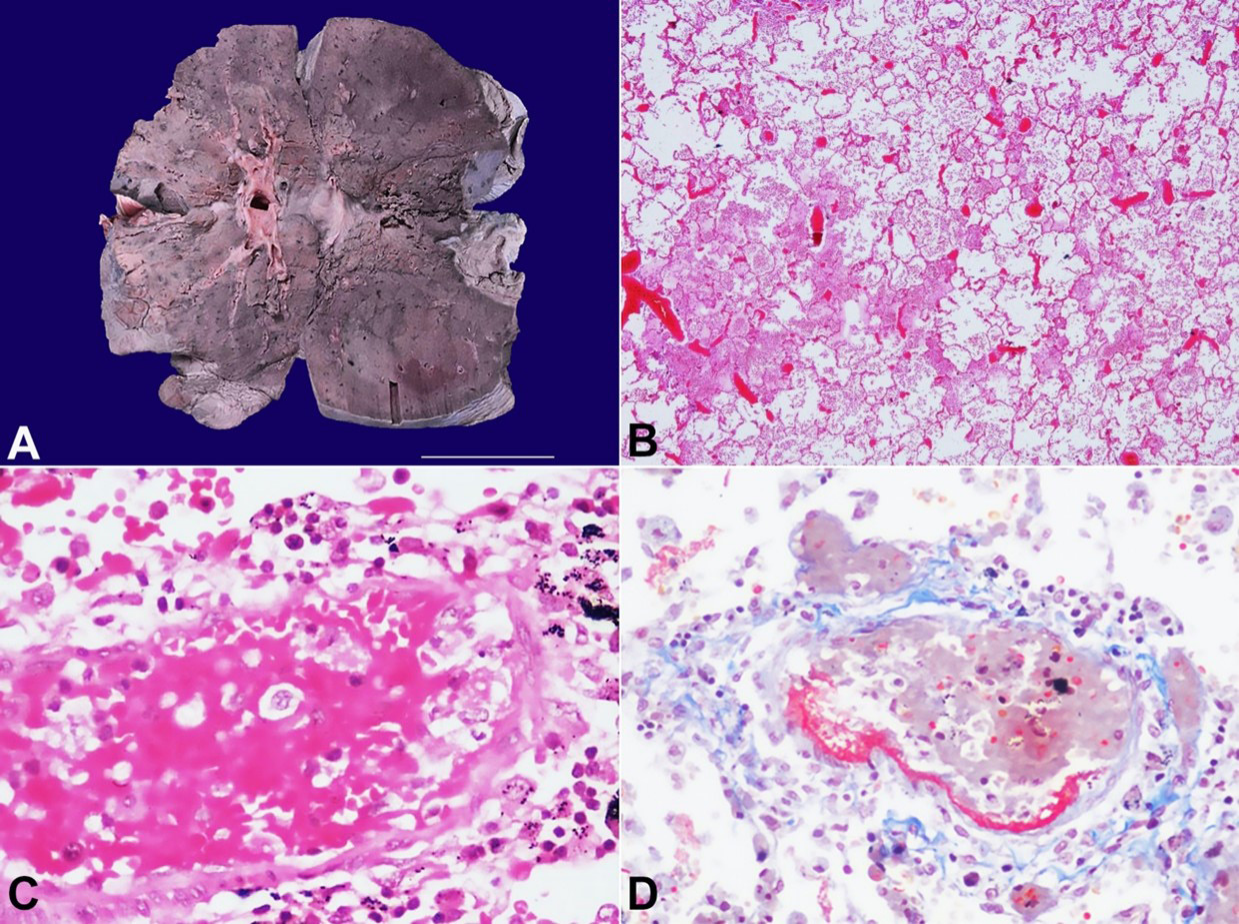
**A –** Gross view of the cut surface of the lung showing focal brownish discoloration. Microphotographs of the lung; **B –** low power examination showing marked vascular congestion with pulmonary edema (H&E, 20X); **C –** pre-acinar pulmonary artery showing marked congestion and evidence of endothelitis in the form of endothelial cell swelling, extravasation of RBCs, and deposition of fibrin material in their wall (H&E, 200x); **D –** Fibrin in the arterial wall is better highlighted on Martius Scarlet blue stain (MSB, 200X).

Both the lungs weighed 1278 g (mRR; 825 g). The pleural surface was smooth and shiny. The cut surface of both lungs was firm and consolidated to feel with areas of hemorrhagic discoloration (Figure 1A). There were no hilar lymphadenopathy and pulmonary thromboembolism. The histologically sections from represented areas showed markedly congested of all pulmonary vasculatures (Figure 1B). Pre-acinar and intra-acinar pulmonary arteries were markedly congested, and there was evidence of endothelitis with the deposition of fibrin material in their wall (Figures 1C and 1D).

**Figure 2:**
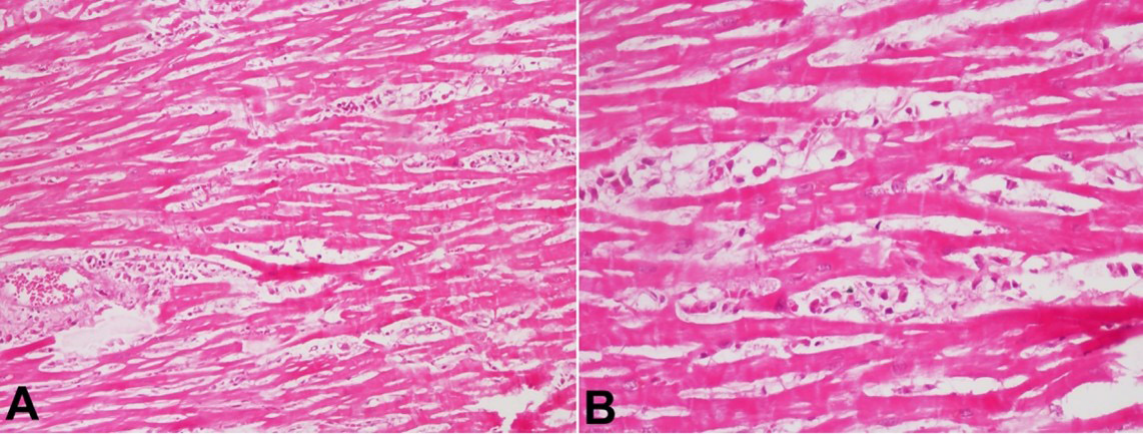
**A –** Photomicrographs of the myocardium showing perivascular and interstitial edema (H&E, 100X); **B –** and mild mixed Inflammatory cell infiltrates (H&E, 200x).

Diffuse alveolar edema and bland pulmonary hemorrhage were also noted. Capillaries in the interalveolar septa also showed significant congestion. Few foci of Bronchopneumonia were noted; however, no organism was identified. The heart weighed 250g (RR, 243 g), and the pericardium showed few foci of pinpoint hemorrhages. All valves and chambers were essentially normal. The histological sections from the heart showed myocarditis with perivascular and interstitial edema (Figure 2); mild to moderate mixed inflammatory cells infiltrates around myocytes, causing its damage. Intramyocardial vessels were markedly congested. The coronaries were dissected and were unremarkable.

**Figure 3:**
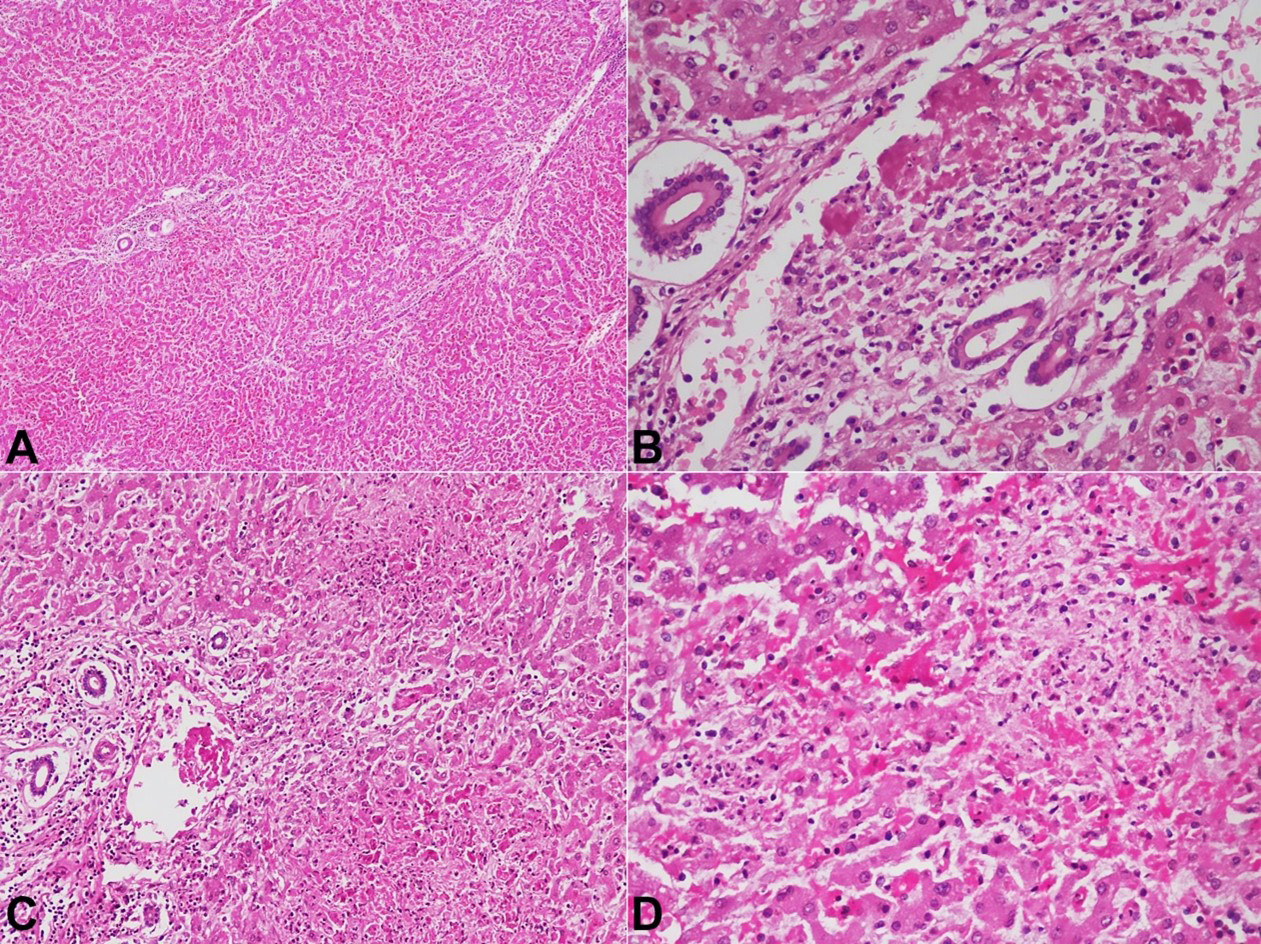
**A –** Photomicrographs of the liver showing maintained lobular architecture with marked sinusoidal dilatation and congestion (H&E, 20X); **B –** Portal tract showing fibrin thrombus in the portal vein with near-complete occlusion of their lumina (H&E, 200x); **C –** Hepatic lobules show multifocal hepatocyte necrosis in Zone 1 (H&E, 200X); **D –** and Zone 2 region (H&E,400X).

The liver weighed 1219 g (RR; 1200-1500) and was grossly unremarkable. The hepatic histopathological sections showed maintained lobular architecture with marked sinusoidal dilatation and congestion (Figure 3A). Portal tracts showed moderate lymphomononuclear inflammatory infiltration. Few portal veins showed evidence of endothelitis with fibrin thrombi (Figure 3B). Multifocal non-zonal confluent hepatocyte necrosis and lobular inflammation were also observed (Figures 3C and 3D).

**Figure 4:**
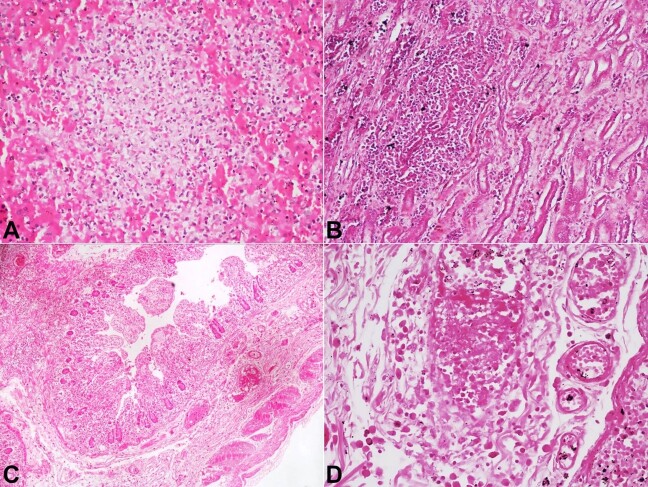
Photomicrographs of: **A –** the spleen showing micro abscess (H&E, 200x); **B –** the kidney showing foci of interstitial nephritis in the medulla (H&E, 200x); **C –** small intestine showing marked vascular congestion and edema in mucosa and submucosa (H&E,100x); **D –** arterioles of intestinal submucosa showing marked congestion with fibrin deposition along the wall indicating vascular injury (H&E, 200x).

The spleen was enlarged and weighed 190 g (RR; 112g). The cut surfaces showed congestion of the red pulp and depletion of the white pulp. Foci of microabscesses were seen (Figure 4A); however, special stains did not reveal any organism. Both kidneys weighed 307 g (RR; 313 g) and were grossly unremarkable. Microscopic examination showed moderate to severe acute tubular injury with few foci of interstitial nephritis at the corticomedullary junction (Figure 4B). The mucosa of both large and small intestines was congested. The** **microscopic examination showed mixed inflammatory cells infiltrates comprising lymphocytes and plasma cells in the lamina propria and marked congestion (Figure 4C). Arterioles in the submucosa showed evidence of endothelitis with deposition of fibrin material along the arterial wall, indicating vascular damage (Figure 4D).

Skeletal muscle, skin, testes, pancreas, stomach, and thyroid were grossly and microscopically unremarkable. Lymph node and bone marrow showed evidence of hemophagolymphohistiocytosis. Section from adrenal gland showed focal adrenalitis.

**Figure 5:**
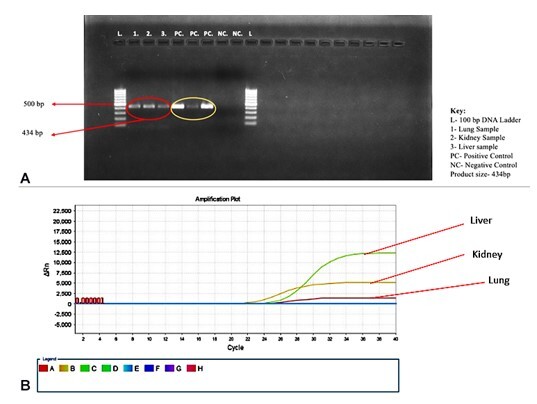
**A –** Gel picture depicting positive 434 bp PCR amplicon of 17kDa gene; **B –** Real-time PCR graph for *OmpA* gene of *Rickettsia*.

Tropical diseases were suspected because of the clinical and histomorphologic features of the index case. Lung, liver, and kidney tissue was initially subjected to PCR for scrub typhus, which was negative. However, because of prominent eschar and high suspicion of rickettsial infection, PCR for panrickettsial gene 17kDa was performed and was positive (Figure 5A). Real-time PCR for *OmpA* gene of *Rickettsia* also came to be positive (Figure 5B). The 434 bp amplified product so obtained was then sequenced using Sanger sequencing. The consensus sequence was prepared using SeqMan (DNASTAR). This consensus sequence was then analyzed using NCBI BLAST, which showed 100% query coverage and 99.6% identity to the gltA gene of *Rickettsia conorii*.

## DISCUSSION

Rickettsial diseases are not an uncommon cause of acute febrile illness in India. Due to a lack of reliable diagnostic tests, RD may be missed in evaluating acute febrile illness. Molecular studies such as nested PCR with specific primers for spotted fever and typhus groups should be performed in suspected cases to increase the detection rate. Using nested PCR, the documented prevalence of RD among acute febrile illness in India was 7%.^2^

The basic pathophysiology of all rickettsial diseases is due to the endotheliotropic infectious nature of its causative bacteria.^1^ After inoculation into the human body from infected arthropods, the bacteria spread through the hematogenous route to different body organs and infect mainly endothelial cells and, to a lesser extent, macrophages. After infection, endothelial cells get activated and stimulate several cell signaling cascades leading to secretions of chemokines and cytokines, particularly interleukins -1β and Tumor Necrosis factor-α.^6^ Further, organisms spread along the local microvasculature network and cause extensive injury.

Histopathological changes in rickettsial diseases result from endothelial injury and increased vascular permeability.^7^ In the index case, marked vascular congestion was observed in nearly all organs examined. The findings such as Diffuse pulmonary edema, interstitial and perivascular edema in the heart, liver, kidneys, and intestines indicate augmented vascular permeability occurred during the disease and caused respiratory distress, hypovolemic shock, and acute kidney injury. Further, the endothelial injury was evident in vasculitis with fibrin material deposition in the microvasculature of the lungs, liver and intestines. Involvement of the liver in the form of portal triaditis with thrombus in the portal vein and multifocal small confluent necrosis of hepatocytes could be the reason for deranged liver function in our case. Hemophagolymphohistiocytosis in the lymph nodes and bone marrow observed in this case was documented in fatal rickettsial disease and might be due to aberrant immune response and cytokine storm.^8^ Histological changes observed in this case are characteristic but not specific for rickettsia diseases. Similar changes can also be seen in other endotheliotropic infectious diseases like scrub typhus and leptospirosis.

## CONCLUSION

Rickettsial disease and other possible endotheliotropic infections should be considered differential diagnoses in evaluating the causes of acute febrile illness with multiorgan involvement and shock. Histological findings such as significant vascular congestion, edema, and vasculitis is characteristic of endotheliotropic infections. On observing such findings in the biopsy done for specific reasons, the pathologist should alert the clinician regarding the possibility of endotheliotropic infection. In suspected cases, molecular methods such as nested PCR using specific primers can be done early for detection and prompt treatment.
